# Pro-Inflammatory and Cytotoxic Effects of Polystyrene Microplastics on Human and Murine Intestinal Cell Lines

**DOI:** 10.3390/biom13010140

**Published:** 2023-01-10

**Authors:** Virginia Mattioda, Valerio Benedetti, Carlotta Tessarolo, Francesca Oberto, Alessandra Favole, Marina Gallo, Walter Martelli, Maria Ines Crescio, Enrica Berio, Loretta Masoero, Alessandro Benedetto, Marzia Pezzolato, Elena Bozzetta, Carla Grattarola, Cristina Casalone, Cristiano Corona, Federica Giorda

**Affiliations:** 1 Istituto Zooprofilattico Sperimentale del Piemonte, Liguria e Valle d’Aosta, Via Bologna 148, 10154 Torino, Italy; 2 ASL 1 Imperiese Via Aurelia Ponente 97, 18038 Sanremo, Italy

**Keywords:** microplastics, polystyrene, polystyrene PS-MPs, HRT-18, CMT-93, gene expression, inflammation, cell-mediated immune response, IL-8, cytotoxicity, environmental pollutants

## Abstract

Plastic is a polymer extremely resistant to degradation that can remain for up to hundreds or thousands of years, leading to the accumulation of massive amounts of plastic waste throughout the planet’s ecosystems. Due to exposure to various environmental factors, plastic breaks down into smaller particles named microplastics (1–5000 μm) and nanoplastics (<1 μm). Microplastics (MPs) are ubiquitous pollutants but, still, little is known about their effects on human and animal health. Herein, our aim is to investigate cytotoxicity, oxidative stress, inflammation and correlated gene modulation following exposure to polystyrene microplastics (PS-MPs) in HRT-18 and CMT-93 epithelial cell lines. After 6, 24 and 48 h PS-MPs treatment, cell viability (MTT) and oxidative stress (SOD) assays were performed; subsequently, expression changes and cytokines release were investigated by Real-Time PCR and Magnetic-beads panel Multiplex Assay, respectively. For each exposure time, a significantly increased cytotoxicity was observed in both cell lines, whereas SOD activity increased only in CMT-93 cells. Furthermore, Magnetic-beads Multiplex Assay revealed an increased release of IL-8 in HRT-18 cells’ medium, also confirmed by gene expression analysis. Results obtained suggest the presence of a pro-inflammatory pattern induced by PS-MPs treatment that could be related to the observed increase in cytotoxicity.

## 1. Introduction

Among the emerging threats affecting humans, animals and ecosystems, environmental pollutants raise very alarming prospects in the near future and place global health seriously at risk [1,2,3,4,5,6,7]. Plastic pollution is one of these; indeed, almost 370 million tons of plastic were produced globally in 2020 alone and, according to the throwaway culture we are living in today, around 40% of total plastic production is for the packaging market, thus increasing plastic pollution [8].

The marine environment is a major sink for plastic pollution that can enter the ocean through land and sea sources. Plastic debris can be frequently found from the sea surface to the seafloor and along the coastline [9].

Plastic is a polymer extremely resistant to degradation that can take up to hundreds and even thousands of years. For example, the general lifetime of plastic bottles ranges from 16 to 48 years [10]; however, experimental studies predicted the life expectancy of plastic bottles under elevated levels of humidity, such as aquatic environments, to be 27 [11] and 93 [12] years. Despite this, the breakdown of large plastic products, due to the exposure to different environmental factors such as UV light and physical abrasion over years, leads to the formation of smaller pieces classified as microplastics (MPs) (size from 1 µm to 5 mm) or nanoplastics (size less than 1 µm) [13,14].

The exposure to environmental MPs by animals and humans can occur via ingestion, inhalation and dermal contact [13,15]. Considering the persistence of this durable polymer in all ecosystems, MPs infiltration in the food chain and consequently in human and animal bodies was frequently reported [9,13,15,16].

For this reason, the scientific community is turning the spotlight on the potential toxic effects these pollutants may have and, in 2016, the European Food Safety Authority (EFSA) published an overview on the presence of MPs in food, reporting a lack of information on the fate of microplastics in the gastro-intestinal tract (GIT) [17].

As the National Reference Center for Marine Mammals Diagnostics (C. Re. Di. Ma.), we engage in research on marine mammals, their environment and the various threats that challenge their survival. Since they are long-lived sentinels that share their trophic chain and habitat with humans, they are used for biomonitoring the health status of the oceans and microplastic pollution [9].

To date, different studies have reported the interaction between MPs and marine mammals [9,18,19], and we investigated the presence and implications of plastic litter in the GIT of stranded cetaceans [20], demonstrating that MPs were the most abundant items of litter isolated after GIT analysis of different cetaceans’ species.

Furthermore, in vivo and in vitro studies concerning the uptake, the cytotoxicity, and the immune and inflammatory response of cells after MPs exposure are present in literature [21,22,23,24,25,26,27,28]. In 2020, Wu and colleagues [25] showed how small-size polystyrene microplastics (PS-MPs) can affect the expression of proliferation and inflammation-related genes in human immortalized Caco-2 intestinal cell line.

The absence of an immortalized intestinal cetacean’s cell line commercially available and the request of EFSA to investigate the effects of MPs in mammalian systems led us to use two different immortalized colorectal adenocarcinoma cell lines: human HRT-18 and murine CMT-93.

In this study, we chose PS-MPs as representative of microplastic particles and investigated their toxic effects on HRT-18 and CMT-93 cell lines by evaluating the possible alteration on inflammatory genes expression through Real-Time PCR technique and the release in the culture medium of inflammatory cytokines by Magnetic Beads Multiplex Assay. Cytotoxicity and oxidative stress were also evaluated by MTT and SOD assay.

## 2. Materials and Methods

### 2.1. Cell Cultures and PS-MPs Treatment, Cell Viability and Oxidative Stress

#### 2.1.1. Cell Cultures and PS-MPs Treatment

Human colorectal epithelial cell line HRT-18 (BS TLC 26, IZSLER, Brescia, Italy) and murine rectal epithelial cell line CMT-93 (BS TLC 14, IZSLER, Brescia, Italy) were maintained in 12-well culture plates CytoOne^®^ (Starlab, Hamburg, Germany) in DMEM medium containing 10% fetal bovine serum (FBS), 1% L-glutamine 200 mM and 1% penicillin/streptomycin 100× and incubated at 37 °C in a humidified atmosphere of 5% CO_2_ and 95% air. Virgin PS-MPs with diameters of 4.8–5.8 µm (Polystyrene Microspheres 1.07 g/cc, Cospheric LLC, Santa Barbara, CA, USA) were suspended in a solution of PBS 1X and 0.1% of Tween 20 to avoid spontaneous aggregation. To prevent PS-MPs from floating in the culture medium, we decided to use polystyrene microspheres characterized by a density higher than water (1.07 g/cc).

HRT-18 and CMT-93 cell lines (seeded 1.5 × 10^6^ cells/mL) were cultured for 24 h until they entered the log phase. Afterward, cells in different wells were exposed to the same concentration of PS-MPs (1 mg/mL) but for a different length of time: 6, 24 and 48 h; untreated HRT-18 and CMT-93 cells were maintained for 48 h as a control group.

During the exposure to PS-MPs, cells were maintained in culture with a conditioned medium without FBS, which could cause PS-MPs aggregation, and without phenol-red to avoid interference in the spectrophotometer reading. All experiments were carried out in triplicate. At the end of each treatment, supernatants were collected and stored in 1.5 mL Eppendorf at −80 °C for Magnetic Beads Multiplex Assay. Cells were washed with PBS 1X and detached using EDTA + Trypsin 0.25% (Gibco ^TM^, Thermo Fisher, Waltham, MA, USA). Finally, cells were harvested in 1.5 mL Eppendorf, resuspended in 500 µL of TRIzol^®^ (Invitrogen-Thermo Fisher Scientific, Waltham, MA, USA) and stored at −80 °C.

#### 2.1.2. Cell Viability—MTT Assay

HRT-18 and CMT-93 cell viability after 6, 24 and 48-h exposure to PS-MPs was assayed using an MTT assay kit (Abcam, ab211091, Cambridge, UK) according to the protocol provided by the manufacturer. Briefly, after medium removal, 50 µL of serum-free media and 50 µL of MTT Reagent were added to each well and cells were incubated for 3 h. Then, 150 µL of MTT Solvent was added to each well and absorbance was assessed at OD590 nm using a SPECTROstar nano^®^ (BMG Labtech, Ortenberg, Germany) microplate absorbance reader. All experiments were carried out in triplicate.

#### 2.1.3. Oxidative Stress—SOD Assay

The Superoxide Dismutase (SOD) Colorimetric Activity Kit (Invitrogen-Thermo Fisher Scientific, Waltham, MA, USA) was used to measure the activity of SOD in cells after different times of PS-MPs exposure according to the manufacturer’s protocol. Briefly, after medium removal, 50 μL of 1X Substrate and 25 µL of 1X Xanthine Oxidase were added into each well and incubated for 20 min at room temperature. Absorbance was assessed at 450 nm using a SPECTROstar nano^®^ (BMG Labtech, Ortenberg, Germany) microplate absorbance reader. All experiments were carried out in triplicate.

### 2.2. Magnetic-Beads Panel MultiplexPlex Assay

Luminex^®^ Performance Assay for the detection of human and murine cytokines (respectively, Biotechne^®^ Human Magnetic Luminex Screening Assay LXSAHM-08 and Biotechne^®^ Mouse Magnetic Luminex Screening Assay LXSAMSM-05, R&D System, Minneapolis, MN, USA) were performed on supernatant samples previously collected from culture plates and stored at −80 °C. Evaluated analytes are listed in Table 1 and Table 2. Briefly, supernatant samples were thawed and centrifuged at 14,000× *g* for 5 min at 4 °C. Samples were diluted 1:2 using Calibrator Diluent RD6-2, which was provided in the kit. All the reagents were prepared following the manufacturer’s protocol. A total 50 µL of Working standards or diluted samples were added to the respective well. Then, 50 µL of reconstructed Microparticle Cocktail was added. The plate was incubated overnight at 4 °C on a shaker at 850× *g*. The day after, the plate was washed using a Bio-Plex^®^ Handheld magnetic washer (Bio-Rad, Hercules, CA, USA) three times using 100 µL of Wash Buffer, and then 50 µL of reconstructed Biotin-Antibody Cocktail was added to the plate and incubated at room temperature for 1 h on a shaker at 850× *g*. Washes have been repeated as before and 50 µL of Streptavidin-PE was added to each well and incubated for 30 min at room temperature on a shaker at 850× *g*. After washing for the last time, the plate was incubated for two minutes and then read using a Bio-Plex 200^®^ plate reader (Bio-Rad, Hercules, CA, USA).

### 2.3. RNA Extraction, Retrotranscription and Quantitative Real-Time PCR

Total RNA from cultured cells was extracted by TRIzol^®^ reagent (Invitrogen-Thermo Fisher Scientific, Waltham, MA, USA) and RNeasy Mini Kit (QIAGEN, Hilden, Germany), then quantified using NanoDrop (ND-2000, Thermo Fisher Scientific, USA). Reverse transcription was performed using 1 µg of the extracted total RNA with a OneScript^®^ Plus cDNA Synthesis kit (Applied Biological Materials, Richmond, BC, Canada) following the manufacturer’s protocol. Relative expression of genes of interest was established by setup of Real-Time PCR reactions with BlasTaq 2X qPCR MasterMix (Applied Biological Materials, Richmond, BC, Canada), cDNA, waters and primers targeted to specific human and murine genes involved in inflammation and proliferation listed in Table 3 (IL-8, IL-18, IL-1β, Cyclin D1 and MAPK1/ERK). All PCR reactions and run conditions applied followed the manufacturer’s specifications. For proper normalization of recorded expression levels, additional assays targeted to reference genes (β-Actin, B2M and HPRT) were included in the study. All primer sets were designed for each target using Primer3 online software [29], further verified and in silico validated by the UCSC Genome Browser (https://genome.ucsc.edu accessed on 11 January 2022).

To evaluate basal stability of all selected transcripts and linearity of PCR conditions, preliminary PCR runs on serial dilutions of cDNA samples from treated and control cell cultures were performed [30,31].

Real-Time PCR runs were performed on a CFX96 Touch Real-Time PCR Detection System (Bio-Rad, Hercules, CA, USA). The relative expression of targeted genes was determined using the comparative threshold cycle (Ct) (2^−∆∆Ct^) method [32].

### 2.4. Statistical Analysis

#### 2.4.1. MTT and SOD Assays Statistical Analysis

MTT assay statistical analysis was performed correlating cytotoxicity effects to the observed absorbance following manufacturer’s instructions. Further statistical analyses were performed using observed absorbance to validate our results. Regarding MTT data, absorbance values—once the blank value of cell-free wells was subtracted—were used. Regarding SOD analysis, the absorbance values obtained from the spectrophotometer reading were converted by interpolating the standard curve with the spectrophotometer readings, as specified in the kit information sheet. A nonparametric regression model was used for all analyses considering the plate variable as a cluster within the model; bootstrap replications (100) were used for the calculate on of standard error and p-value. The same model was used for trend assessment when possible. All these analyses were performed using STATA software (version 17).

#### 2.4.2. Magnetic-Beads Panel MultiplexPlex Assay Statistical Analysis

Bio-Plex Manager^TM^ software (Bio-Rad, Hercules, CA, USA) automatically calculated cytokine concentrations using a standard curve derived from a recombinant cytokine standard following manufacturer’s instructions.

#### 2.4.3. Real-Time PCR Statistical Analysis

Relative quantification (RQ) data of targeted cDNAs were calculated with CFX Maestro Software (version 2.2, Bio-Rad, Hercules, CA, USA). The software allowed to verify significance of differential expression in all analyzed genes by one-way ANOVA with Tukey’s post hoc test (*p* < 0.05). All reported statistical analyses were performed using three repeats per time for each biological replicate.

## 3. Results

### 3.1. Cell Cultures, Cell Viability and Oxidative Stress after PS-MPs Treatment

**Figure 1:**
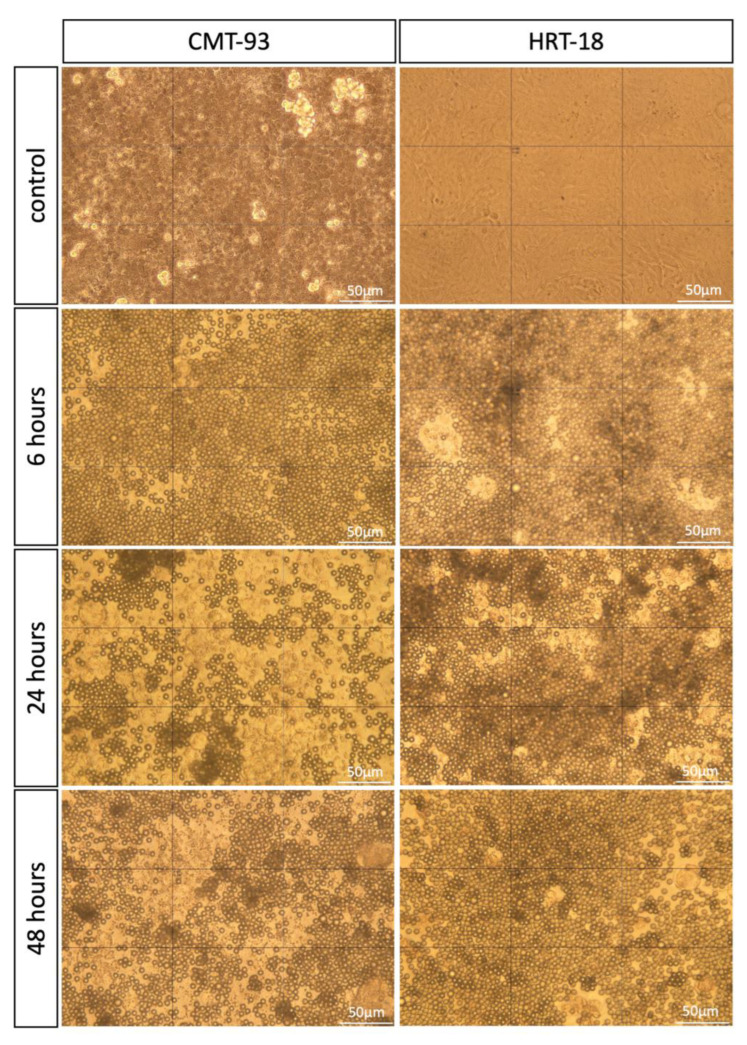
Light microscopy images of cell cultures (CMT-93 and HRT-18) treated with 1 mg/mL round-shaped PS-MPs for 6, 24, 48 h and control group. All images were taken at 40× magnification. Scale bar = 50 μm.

The cultured HRT-18 and CMT-93 cell lines were exposed to the same concentration of PS-MPs (1 mg/mL) at different times of exposure (6, 24 and 48 h). After treatment, the placement of PS-MPs in culture plates was assessed using a light microscope to ensure that MPs did not float and stay in contact with cells (Figure 1).

Cell viability after PS-MPs exposure was assessed via MTT analysis and data were evaluated both considering the cytotoxicity rate (100 × (control − samples)/control) following manufacturer’s instructions and using a nonparametric regression model on the absorbance data obtained to perform statistical analysis.

**Figure 2:**
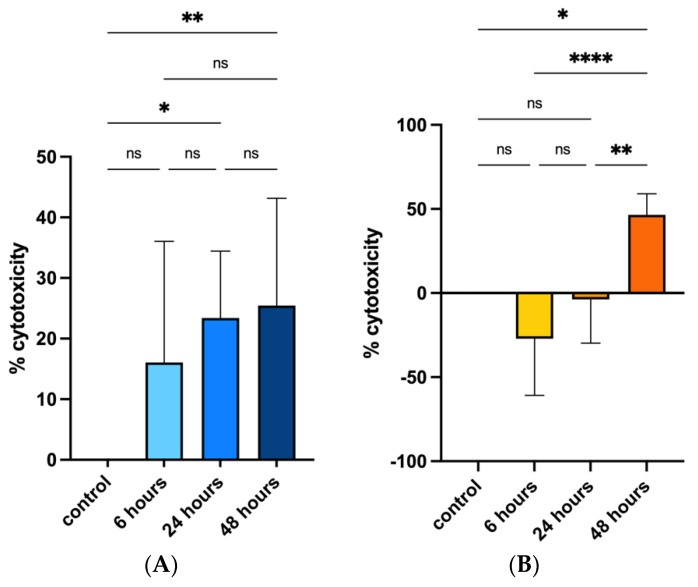
Effects of PS-MPs treatment on cytotoxicity in CMT-93 (**A**) and HRT-18 (**B**), respectively. Cytotoxicity was evaluated both in control group and 6, 24 and 48 h after 1 mg/mL PS-MPs treatment via MTT assay. Percentage was evaluated using cytotoxicity rate (100 × (control − samples)/control). The error bars indicate Standard Deviation. **** *p*-value ≤ 0.0001; ** *p*-value ≤ 0.01; * *p*-value ≤ 0.05; ns *p*-value > 0.05.

**Figure 3:**
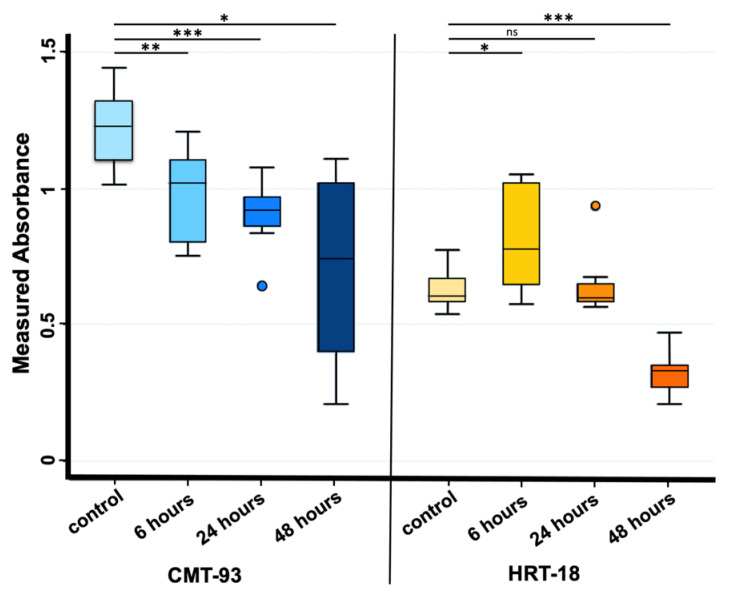
Absorbance values observed at OD590 nm in CMT-93 (**left**) and HRT-18 (**right**) cell cultures measured both in the control group and 6, 24 and 48 h after 1 mg/mL PS-MPs treatment via MTT assay. *** *p*-value ≤ 0.001; ** *p*-value ≤ 0.01; * *p*-value ≤ 0.05; ns *p*-value > 0.05. Regression model analysis showed a statistically significant trend in CMT-93 cells (**left**) considering controls and all exposure times (beta value: −0.011, CI: −0.018 −0.006).

Cytotoxicity rate showed increasing values only for the CMT-93 cell line rising from 18.4% at 6 h of exposure, to 24.9% at 24 h and 42.8% at 48 h compared with the control group (Figure 2A); regression model analysis confirmed these data with a statistically significant trend (beta value: −0.011, CI: −0.018 −0.006) considering controls and all exposure times (6, 24 and 48 h) (Figure 3).

Cytotoxicity rate in the HRT-18 cell line was increased between the control and 48 h exposure groups (Figure 2B); regression model analysis of absorbance raw data confirmed the significant difference (Figure 3).

These analyses suggested that PS-MPs treatment induced an inhibitory effect on cell viability compared with controls both in human and murine cell lines, additionally showing a significant up-trend between exposure times and cytotoxicity in murine cell lines.

**Figure 4:**
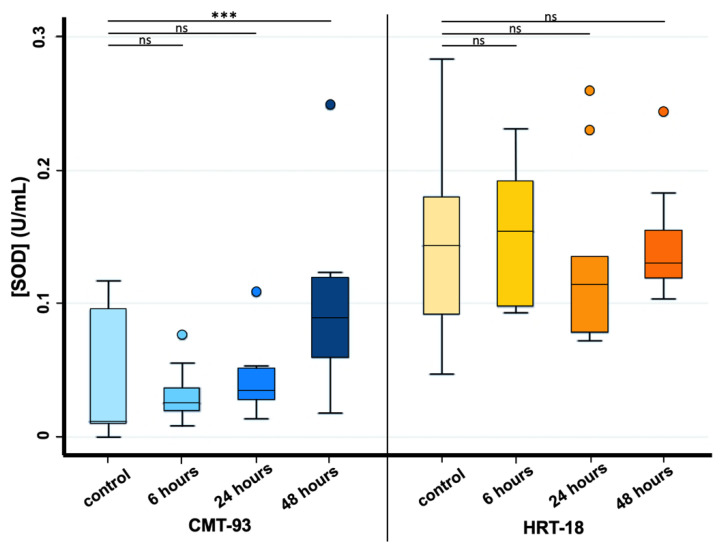
Effects of PS-MPs treatment on SOD activity in CMT-93 (**left**) and HRT-18 (**right**). SOD activity was evaluated both in the control group and 6, 24 and 48 h after 1 mg/mL PS-MPs treatment via SOD assay. *** *p*-value ≤ 0.001; ns *p*-value > 0.05.

Considering SOD analysis, a statistically significant increase in SOD activity was observed only between controls and 48 h exposure time in CMT-93 cell lines, whereas for HRT-18 cell lines this statistical significance was not found for any exposure time due to the presence of an outlier value among the control group (Figure 4).

### 3.2. Magnetic-Beads Panel MultiplexPlex Luminex Assay

Biotechne^®^ Human (LXSAHM-08) and Mouse Magnetic Luminex Screening Assay (LXSAMSM-05) were performed, respectively, on HRT-18 and CMT-93 cell lines exposed to PS-MPs (1 mg/mL) for 6, 24, 48 h and controls.

**Figure 5:**
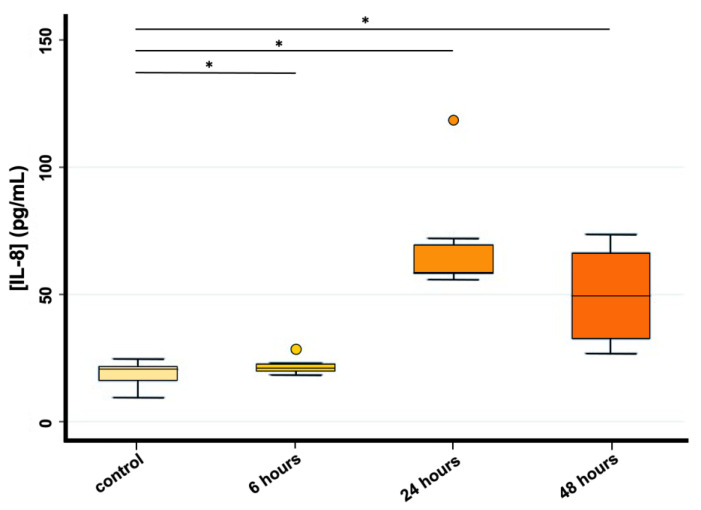
IL-8 concentration (pg/mL) evaluated through Magnetic-beads Assay Luminex in HRT-18 cell lines (* *p* < 0.05) both in the control group and 6, 24 and 48 h after PS-MPs exposure (1 mg/mL).

IL-8 concentration analysis performed on HRT-18 cells showed a statistically significant difference between controls and all exposure times (*p* < 0.05), indicating an increased release of IL-8 in the medium of HRT-18 cells 6, 24 and 48 h after PS-MPs exposure (1 mg/mL). No linear trend was found; the concentration of IL-8 showed a peak at 24 h and then decreased again in the 48 h exposure (Figure 5). Unfortunately, IL-8 concentration analysis was performed only on HRT-18 data and not on CMT-93 due to the infeasibility to include this cytokine in the murine assay.

Furthermore, among the other cytokines investigated—IL-1β, IL-6, IL-7, IL-10, IL-15, IL-18, IL-23 and IL-33 evaluated in HRT-18 culture medium (Table 1) and IL-1β, IL-6, IL-7, IL-10 and IL-33 evaluated in CMT-93 culture medium (Table 2)—no statistically significant differences were detectable between 6, 24 and 48 h exposure compared with controls. Most of the obtained values fit under the lowest point of the standard curve; these readings could not be taken to account for statistical analysis.

### 3.3. Quantitative Real-Time PCR

Quantitative Real-Time PCR analysis was performed on murine and human samples after PS-MPs treatment and relative controls. The selected primers’ sequences and their specifications are listed in Table 3.

The β-Actin and Cyclin D1 were chosen as reference genes, being characterized by stable expression in both PS-MPs treated and control samples, as verified by Genorm analysis embedded in Maestro Software (Biorad) [33].

**Figure 6:**
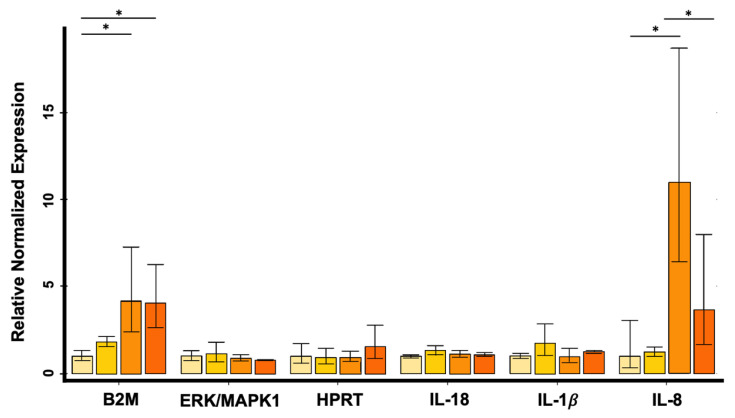
Relative expression levels of B2M, MAPK1/ERK, HPRT, IL-18, IL-1β and IL-8 in HRT-18 cell line evaluated by quantitative Real-Time PCR (* *p* < 0.05). Data were reported in linear scale. Cyclin D1 and β-Actin were used as reference genes for normalization. The error bars indicate Standard Deviation.

Subsequently, RQ analysis performed on HRT-18 cell line showed up-regulation of the IL-8 gene 6, 24 and 48 h after exposure to PS-MPs compared with untreated controls (Figure 6); the increase resulted to be statistically significant between the control group and 24 h exposure group (*p*-value = 0.0173) and between 6 and 24 h (*p*-value = 0.0280), respectively.

Furthermore, B2M was up-regulated at 24 and 48 h after exposure compared with controls (24 h vs. control group *p*-value = 0.0079; 48 h vs. control group *p*-value = 0.0088) (Figure 6). No differences were observed at the expression level of human IL-18, IL-1β, MAPK1/ERK and HPRT genes (Figure 6).

In CMT-93 cells, no alterations were observed in the expression levels of IL-8/CXCL8, IL-18, IL-1β, MAPK1/ERK, B2M and HPRT genes after PS-MPs exposure.

## 4. Discussion

According to the Worldwatch Institute, about 10 to 20 million tons of plastic enters aquatic ecosystems every year, exposing a global issue that affects all ecosystems and consequently the complete food chain [5,9]. MPs pollution has now been found in the most remote places on Earth, from Antarctic’s snow [34] to central Asian deserts [35] and lagoons of French Polynesia [36]. Moreover, these particles can be found in everyday life such as in bottled water, air and our own bodies [37,38,39,40,41]. Therefore, the current situation requires urgent action and cooperation for deeper knowledge on the hazard that plastic debris could bring to human, animal, and plant health in view of a One Health approach.

Several previous in vitro and in vivo studies have highlighted the effects induced by these ubiquitous pollutants on living organisms, investigating their implications on cell viability, oxidative stress, membrane integrity, gene expression and immune response [22,23,24,25,26,27,28,42,43].

Ingestion of pollutants such as MPs or most common heavy metals from contaminated food and water is the primary source of exposure to contaminants [44,45] that could induce severe damage to animal and human health [3,4]. Previous studies demonstrated that MPs could interact with intestinal cells [23,42]; for this reason, we decided to use two immortalized intestinal epithelial cell lines from two different mammalian species.

When choosing the nature of microparticles, we opted for polystyrene microbeads, since they have been employed in several previous studies [23,25,42] and, unlike polyethylene, which has been equally used, did not float in the medium [27]; considering shape, spherical particles were used due to commercial availability.

MPs size plays a significant role since smaller particles are easily absorbed through the intestinal barrier impacting on cellular health [46]; in the current study, we took as references two previous studies that used the same polymer, concentrations and exposure times on two different cell lines, obtaining similar results; in fact, after 24 and 48 h exposure, smaller PS-MPs (1, 1.72 and 4 µm) showed reduced cell viability both in human epithelial colorectal adenocarcinoma (Caco-2) and lung bronchial epithelial (BEAS-2B) cells, respectively [26,47]. According to these results, we decided to use particles that approached this size range (4.8–5.8 µm).

The MPs concentration range is quite varied in literature; however, according to previous studies [23,26,47,48], major cell alterations were reported when a higher concentration of microplastics was used: based on the average of concentration ranges used by other authors, we chose to investigate the effects of a single MP’s concentration (1 mg/mL) exposure over different periods of time. Considering that the digestion process takes about 6 h and that the average exposure time in literature lasts from 6 to 48 h, with a maximum 72 h [23,25,49], we decided to expose both cell lines to PS-MPs for 6, 24 and 48 h.

In the present study, both cell lines exposed to PS-MPs showed an increase in cytotoxicity between controls and treated cells at 48 h of exposure. In addition, CMT-93 cell lines showed a statistically significant upward trend to be interpreted as an increase in cytotoxicity resulting from the increased contact time of the culture cells with the microplastics. The reduced cell viability after MPs exposure was previously reported in literature supporting our results [24,26,42,49]; conversely, other studies did not find such an increase in cytotoxicity [23,43,50].

In literature, the cytotoxicity of MPs is frequently attributed to membrane damage and oxidative stress [51]; notably, loss of membrane integrity causes pore formation in the membrane and consequent reactive oxygen species (ROS) generation from the mitochondria, which, in turn, increase within the cell causing mitochondrial damage with consequent release of pro-apoptotic factors and production of pro-inflammatory cytokines that could lead to the activation of cell death pathways [46]. All the mechanisms mentioned above may contribute to the decreased cell viability observed here.

In the current study, we decided to evaluate oxidative stress after 6, 24 and 48 h MPs exposure by referring to increased SOD activity in cells; we achieved a statistically significant increase only between controls and 48 h exposure in CMT-93 cell lines, whereas, for human HRT-18 cell lines, this statistical significance was not found for any exposure. Several studies have shown a link between ROS production and MPs exposure: Dong and colleagues [26] showed a significant increase in BEAS-2B cells exposed to high-dose PS-MPs for 24 h; Wu and colleagues [23] obtained increased SOD activity after 48 h exposure compared to the control group; and Wang and colleagues [42] reported a significant increase in ROS levels after 120 µg/mL PS-MPs exposure.

Conditions that lead to tissue damage following MPs exposure may be facilitated by ROS translocation into the nucleus, which cause DNA damage and contribute to a genotoxic effect [52] with inflammation being the early reaction to cell damage. Considering this, we decided to further investigate local effects on the cell-mediated immune response that may result from the contact between cells and MPs [53,54] by evaluating cytokines release in culture medium and changes in gene expression.

By referring to the previous study of Wu and colleagues [25], we considered different genes, including those involved in inflammation and proliferation, to evaluate gene modulation after MPs treatment. Compared with the previous study [25], we pursued evaluating the release of a panel of pro-inflammatory cytokines in the culture medium to analyze the inflammatory response.

In this work, Magnetic-beads panel Multiplex Assay revealed significant differences among controls and all exposure times, indicating an increased release of IL-8 in the medium of HRT-18 cells 6, 24 and 48 h after treatment with a peak at 24 h. This result was confirmed by Real-Time PCR showing the same non-linear trend observed. Considering the up-regulation of IL-8 in HRT-18 cell lines, we obtained consistent results with the previous study of Wu and colleagues [25], which revealed that 5 µm PS-MPs had a highly significant effect on gene expression even at lower concentrations (12.5–50 mg/L) compared with those used in our study. Furthermore, Dong and colleagues [26] by using ELISA technique also showed an increased release of IL-8 in BEAS-2B cells exposed to the same PS-MPs concentration used in our study. Conversely, Lehner and colleagues [43] reported no significant release of IL-8 after 6, 24 and 48 h exposure to different MPs polymers using doses of up to 1380.0 µg/cm^2^ in a 3D intestinal model.

IL-8 is an important chemokine involved in neutrophil recruitment during the acute inflammatory response, which plays similar roles in humans and mice; in particular, IL-8 (also called CXCL-8) has important functions as a chemotactic factor by attracting neutrophils, basophils and T-cells to clear pathogens and protect the host from infection during inflammation in humans [55,56]. The murine homologue IL-8/CXCL-8 is involved in hematopoiesis, inflammatory response and neutrophil chemotaxis.

In conclusion, the up-regulation of IL-8 and its increased release in the culture media after PS-MPs treatment suggest the presence of a pro-inflammatory pattern induced by the contact between cells and PS-MPs that could be related to the observed increase in cytotoxicity, in agreement with other studies performed on different cell lines [25,26,42]. This PS-MPs-induced IL-8 inflammatory response, if persistent, could be amplified and contribute to several pathophysiological processes in the GIT, such as chronic inflammatory states and gastric and colonic carcinomas [26,57].

It should be taken into account that the use of different cell lines, MPs polymers, concentrations and exposure times reported in literature make different outcomes inevitable. Furthermore, it is important to keep in mind that conditions described in the literature for in vitro studies still suffer from limitations such as the use of sterile microplastics and cells of a cancerous origin; this means that results obtained must be carefully interpreted since they do not necessarily correspond to what is found in nature [58,59].

Although, to the best of our knowledge, this is the first in vitro study describing PS-MPs effects in human colorectal carcinoma intestinal cell line HRT-18 and providing further information about IL-8 modulated expression and release after PS-MPs treatment, further studies that are more representative of natural conditions will be needed to shed further light on microplastic toxicity.

## 5. Conclusions

This study furthers our understanding of how MPs can affect cell viability, SOD activity, gene expression and inflammation in human and murine intestinal cell lines. Although not the first in demonstrating MPs-induced cytotoxicity and immunological alterations, the present study confirmed the decrease in cell viability in both intestinal cell lines after PS-MPs treatment in a time-dependent manner and showed increased SOD activity in CMT-93. In HRT-18, the up-regulation of IL-8 gene goes along with the rise of IL-8 in the supernatant, suggesting an inflammatory state induced by PS-MPs exposure.

These findings clearly show the potential of PS-MPs to affect cellular viability, providing important baseline information for future research on MPs effects on human and animal health.

## Figures and Tables

**Table 1:** Details of Human Magnetic Luminex Screening Assay (9-Plex) LXSAHM-08.

Analyte	Bead Region	Bead Region	Standard Curve (pg/mL)	Cell Culture	Sensitivity (pg/mL)
IL-1β/IL-1F2	28	A	17.7–4300	1:2	0.8
IL-6	13	A	4.53–1100	1:2	1.7
IL-7	29	K	5.14–1250	1:2	0.410
IL-8/CXCL8	18	A	4.12–1000	1:2	1.8
IL-10	22	A	4.12–1000	1:2	1.6
IL-15	63	J	6.3–1550	1:2	1.01
IL-18/IL-1F4	78	C	7.12–1730	1:2	1.93
IL-23	76	C	144–35,000	1:2	11.4
IL-33	14	C	12.3–3000	1:2	1.8

**Table 2:** Details of Mouse Magnetic Luminex Screening Assay (5-Plex) LXSAMSM-05.

Analyte	Bead Region	Bead Region	Standard Curve (pg/mL)	Cell Culture	Sensitivity (pg/mL)
IL-1β/IL-1F2	19	Mouse A	247–60,000	1:2	41.8
IL-6	27	Mouse A	28.8–7000	1:2	2.30
IL-7	14	Mouse C	267–65,000	1:2	35.4
IL-10	28	Mouse A	12.8–3100	1:2	8.20
IL-33	43	Mouse B	82.3–20,000	1:2	57.1

**Table 3:** Primers used for quantitative Real-Time PCR analysis of human and murine genes.

Gene	Reference Number	Forward Sequence (5′-3′)	Reverse Sequence (5′-3′)	Species
IL-8	NM_001310420.1	CTCTTGGCAGCCTTCCTGAT	TTTGGGGTGGAAAGGTTTGGA	Human
IL-18	NM_001386420.1	AAGATGGCTGCTGAACCAGT	TGCCAAAGTAATCTGATTCCAGG	Human
IL-1β	NM_000576.3	TCGCCAGTGAAATGATGGCT	GGTCGGAGATTCGTAGCTGG	Human
Cyclin D1	NM_053056	AGCTGTGCATCTACACCGAC	GAAATCGTGCGGGGTCATTG	Human
MAPK1/ERK	NM_002745.5	CGTGTTGCAGATCCAGACCA	GCCAGAATGCAGCCTACAGA	Human
β-Actin	NM_001101.5	ACAGAGCCTCGCCTTTGC	CGCGGCGATATCATCATCCA	Human
B2M	NM_004048.4	CTGCCGTGTGAACCATGTGA	TCAAACCTCCATGATGCTGC	Human
HPRT	NM_000194.3	TGCTGAGGATTTGGAAAGGGT	GGGCTACAATGTGATGGCCT	Human
IL-8/CXCL8	NM_011339.2	TGATGCTCCATGGGTGAAGG	CAGAAGCTTCATTGCCGGTG	Murine
IL-18	NM_001357221.1	GGCTGCCATGTCAGAAGACT	ACAGTGAAGTCGGCCAAAGT	Murine
IL-1β	NM_008361.4	GCCACCTTTTGACAGTGATGAG	GACAGCCCAGGTCAAAGGTT	Murine
Cyclin D1	NM_001379248.1	AAACAAGGACCCCCTCCATC	GGCTTCAATCTGTTCCTGGC	Murine
MAPK1/ERK	NM_001038663.1	CCTCCTGCTGAACACCACTT	ATCTGGATCTGCAACACGGG	Murine
β-Actin	NM_007393.5	CTGTCGAGTCGCGTCCACC	CGCAGCGATATCGTCATCCAT	Murine
B2M	NM_009735.3	GAGCCCAAGACCGTCTACTG	GGTTCAAATGAATCTTCAGAGCATC	Murine
HPRT	NM_013556.2	TTCTTTGCTGACCTGCTGGA	TTATGTCCCCCGTTGACTGA	Murine

## Data Availability

The data presented in this study are available within the article.

## Abbreviations

MPs	Microplastics
PS-MPs	Polystyrene microplastics
GIT	Gastro-intestinal tract
EFSA	European Food Safety Authority
CREDIMA	National Reference Center for Marine Mammals Diagnostics
MTT	3-(4,5-dimethylthiazol-2-yl)-2,5-diphenyl-2H-tetrazolium bromide
SOD	Superoxide dismutase
ROS	Reactive oxygen species
